# Exploring the mechanism of curcumin in the treatment of colon cancer based on network pharmacology and molecular docking

**DOI:** 10.3389/fphar.2023.1102581

**Published:** 2023-02-15

**Authors:** Qingmin He, Chuan Liu, Xiaohan Wang, Kang Rong, Mingyang Zhu, Liying Duan, Pengyuan Zheng, Yang Mi

**Affiliations:** ^1^ Henan Key Laboratory of Helicobacter Pylori and Microbiota and Gastrointestinal Cancer, Marshall B. J. Medical Research Center of Zhengzhou University, The Fifth Affiliated Hospital of Zhengzhou University, Zhengzhou, Henan, China; ^2^ Academy of Medical Science, Zhengzhou University, Zhengzhou, Henan, China; ^3^ Department of Gastroenterology, Renmin Hospital of Wuhan University, Wuhan, Hubei, China; ^4^ Department of Gastroenterology, The Fifth Affiliated Hospital of Zhengzhou University, Zhengzhou, Henan, China

**Keywords:** molecular docking, network pharmacology, curcumin, colon cancer, immune infiltration, molecular mechanism

## Abstract

**Objective:** Curcumin is a plant polyphenol extracted from the Chinese herb turmeric. It was found that curcumin has good anti-cancer properties in a variety of cancers, but the exact mechanism is not clear. Based on the network pharmacology and molecular docking to deeply investigate the molecular mechanism of curcumin for the treatment of colon cancer, it provides a new research direction for the treatment of colon cancer.

**Methods:** Curcumin-related targets were collected using PharmMapper, SwissTargetPrediction, Targetnet and SuperPred. Colon cancer related targets were obtained using OMIM, DisGeNET, GeneCards and GEO databases. Drug-disease intersection targets were obtained *via* Venny 2.1.0. GO and KEGG enrichment analysis of drug-disease common targets were performed using DAVID. Construct PPI network graphs of intersecting targets using STRING database as well as Cytoscape 3.9.0 and filter core targets. Molecular docking *via* AutoDockTools 1.5.7. The core targets were further analyzed by GEPIA, HPA, cBioPortal and TIMER databases.

**Results:** A total of 73 potential targets of curcumin for the treatment of colon cancer were obtained. GO function enrichment analysis yielded 256 entries, including BP(Biological Progress):166, CC(celluar component):36 and MF(Molecular Function):54. The KEGG pathway enrichment analysis yielded 34 signaling pathways, mainly involved in Metabolic pathways, Nucleotide metabolism, Nitrogen metabolism, Drug metabolism - other enzymes, Pathways in cancer,PI3K-Akt signaling pathway, etc. CDK2, HSP90AA1, AURKB, CCNA2, TYMS, CHEK1, AURKA, DNMT1, TOP2A, and TK1 were identified as core targets by Cytoscape 3.9.0. Molecular docking results showed that the binding energies of curcumin to the core targets were all less than 0 kJ-mol-1, suggesting that curcumin binds spontaneously to the core targets. These results were further validated in terms of mRNA expression levels, protein expression levels and immune infiltration.

**Conclusion:** Based on network pharmacology and molecular docking initially revealed that curcumin exerts its therapeutic effects on colon cancer with multi-target, multi-pathway. Curcumin may exert anticancer effects by binding to core targets. Curcumin may interfere with colon cancer cell proliferation and apoptosis by regulating signal transduction pathways such as PI3K-Akt signaling pathway,IL-17 signaling pathway, Cell cycle. This will deepen and enrich our understanding of the potential mechanism of curcumin against colon cancer and provide a theoretical basis for subsequent studies.

## 1 Introduction

Colon cancer (CC) has the third highest incidence of all tumors worldwide and is a common cause of oncologic death (Bray et al., 2018). Meanwhile, the incidence and morbidity and mortality of CC are increasing rapidly. The development of CC is a long-term complex process, and although the diagnosis and treatment measures of CC have made great progress in recent years, the 5-year survival rate of CC is still less than 40%, and most CC patients develop tumor recurrence, metastasis and drug resistance (Riihimäki et al., 2016). With the development of herbal medicine in recent years, the monomeric active ingredients extracted from Chinese herbs have become a hot spot for global research. The Chinese medicinal ingredient curcumin is a biologically active substance extracted from the rhizome of turmeric, which belongs to acidic polyphenolic compounds (Zang et al., 2014). A large number of studies have shown that curcumin has good clinical application value as it inhibits tumor cell activity, reduces migration invasion ability, induces autophagy and promotes apoptosis (Doello et al., 2018; Shakeri et al., 2019). In recent years, the potential anticancer properties of curcumin have attracted more attention. However, the anti-cancer mechanism of curcumin is not fully understood.

Chinese medicine has multi-target and multi-pathway mechanisms of action for treating diseases. Therefore, it is necessary to use big data to mine all existing targets and pathways related to curcumin and CC. Network pharmacology is a method that integrates bioinformatics and pharmacology. Through data integration and computational analysis, it can systematically clarify the relationship between drugs and diseases and explore the mechanism of drug action (Boezio et al., 2017). In this study, we screened and predicted the potential targets and signaling pathways of curcumin for the treatment of CC by means of network pharmacology and molecular docking techniques, and provided scientific basis for later drug development and clinical application.

## 2 Materials and methods

### 2.1 Database and research process

**FIGURE 1:**
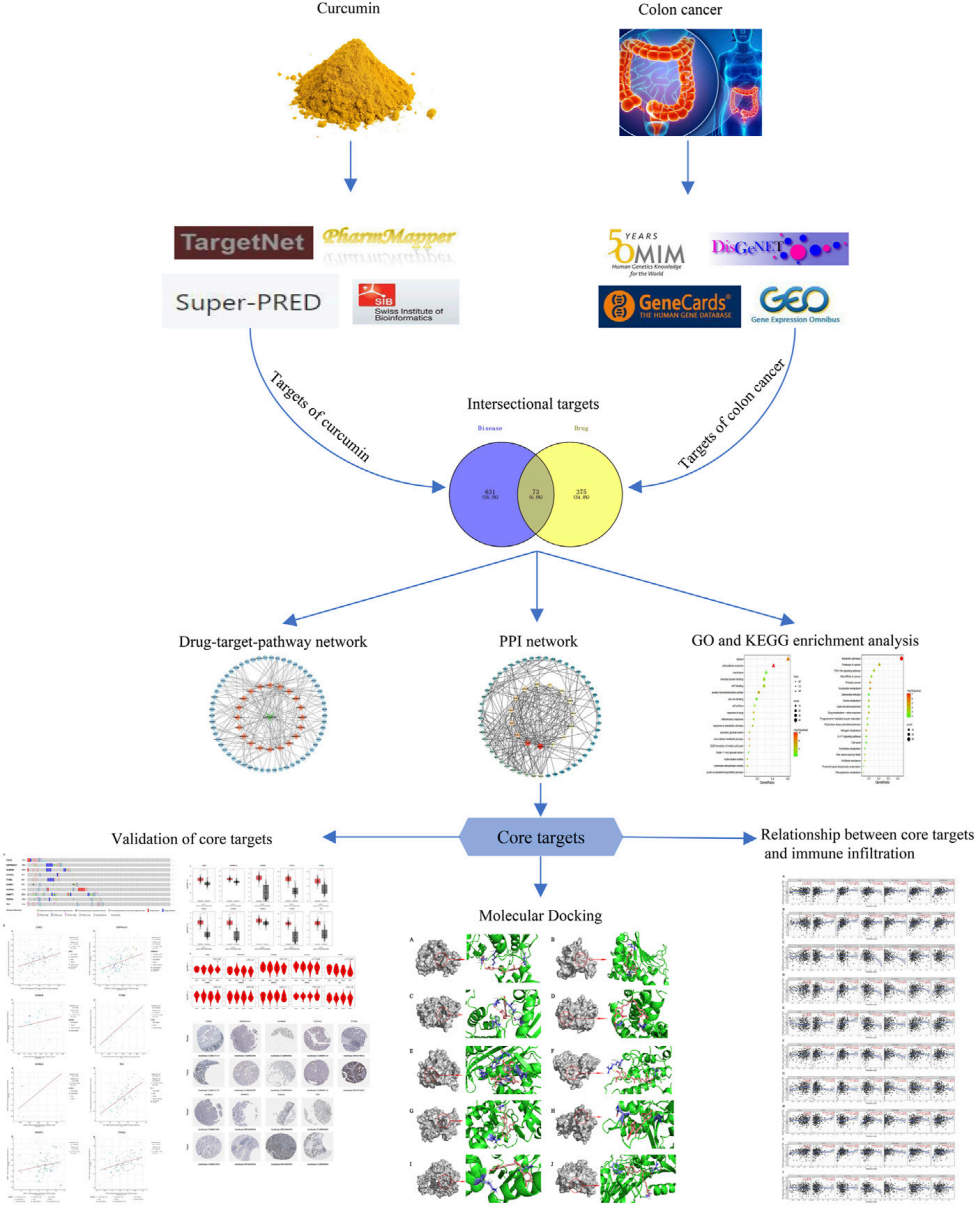
This study is a detailed flow chart of a network-based pharmacology study.

The databases involved in this study (Table 1) and the research process outline (Figure 1).

**TABLE 1:** Basic information of the database used for the screening of curcumin in the treatment of colon cancer.

Name	URL
PubChem	https://pubchem.ncbi.nlm.nih.gov/
PharmMapper	http://lilab-ecust.cn/pharmmapper/
SuperPred	https://prediction.charite.de/
Targetnet	http://targetnet.scbdd.com/
SwissTargetPrediction	http://www.swisstargetprediction.ch/
GEO	https://www.ncbi.nlm.nih.gov/geo/
OMIM	https://www.omim.org/
GeneCards	https://www.genecards.org/
DisGeNET	https://www.disgenet.org/
GEPIA	http://gepia.cancer-pku.cn/
HPA	https://www.proteinatlas.org/
CBioPortal	https://www.cbioportal.org/
TIMER	https://cistrome.shinyapps.io/timer/
Uniprot	https://www.uniprot.org/
STRING	https://cn.string-db.org/
RCSB PDB	https://www.rcsb.org/
Bioinformatics	http://www.bioinformatics.com.cn/
DAVID	https://david.ncifcrf.gov/
Venny 2.1.0	https://bioinfogp.cnb.csic.es/tools/venny/index.html
KEGG Mapper	https://www.kegg.jp/kegg/mapper/
Chemsrc	https://www.chemsrc.com/

### 2.2 Access to potential targets of curcumin

Details of curcumin are available in PubChem (https://pubchem.ncbi.nlm.nih.gov/) (Kim et al., 2021) using CAS: 458-37-7 as the search term. Obtain the targets of curcumin using curcumin’s SDF file or curcumin’s Canonical SMILES in PharmMapper (http://lilab-ecust.cn/pharmmapper/), SwissTargetPrediction (http://www.swisstargetprediction.ch/), Targetnet (http://targetnet.scbdd.com/) and SuperPred (https://prediction.charite.de/) (Nickel et al., 2014; Yao et al., 2016; Wang et al., 2017; Daina et al., 2019). The obtained targets were translated into gene names through the UniProt database (https://www.uniprot.org/) (UniProt Consortium, 2018). Finally, the obtained target genes were combined and de-duplicated. The results were the potential targets of curcumin.

### 2.3 CC-related target collection

GSE74602 was selected from the GEO database (https://www.ncbi.nlm.nih.gov/geo/) as the study subject (Clough and Barrett, 2016). Differentially expressed genes were screened using GEO2R with adj.P.Val < 0.05, |logFC|>1 as screening criteria. The obtained differentially expressed genes were presented in volcano plots by TBtools (Chen et al., 2020). Some CC-related targets were obtained by searching the OMIM (https://www.omim.org/), DisGeNET (https://www.disgenet.org/), and GeneCards databases (https://www.genecards.org/) (Amberger et al., 2015; Stelzer et al., 2016; Piñero et al., 2020) using the search term “colon cancer”. The differentially expressed genes obtained from the GEO database were intersected with the CC-related targets obtained from these three databases to obtain CC disease-related targets.

### 2.4 Drug-disease common target screening and PPI network construction

The intersection of curcumin targets and CC targets was performed using Venny 2.1.0 (https://bioinfogp.cnb.csic.es/tools/venny/index.html), and the intersecting genes represented the potential targets of curcumin for CC treatment. The protein-protein interaction network of common targets was constructed using STRING database (https://cn.string-db.org/), and the tsv file was downloaded and imported into Cytoscape 3.9.0 software for visualization.

### 2.5 GO and KEGG enrichment analysis

The GO and KEGG enrichment analysis of potential targets of curcumin for CC treatment was performed by “Functional Annotation” in the DAVID website (https://david.ncifcrf.gov/) (Sherman et al., 2022). The obtained data were organized and visualized by Bioinformatics (http://www.bioinformatics.com.cn/).

### 2.6 Drug-target-pathway network construction

The drug-target-pathway network was constructed by introducing curcumin, potential targets of curcumin for CC treatment and KEGG pathway into Cytoscape. The nodes represent curcumin, genes or pathways, and the connecting lines represent the relationship of biomolecules.

### 2.7 Core targets screening

In “cytohubba” of Cytoscape 3.9.0. Degree, Maximum Neighborhood Component (MNC), Maximal Clique Centrality (MCC) and Closeness were used to filter the top 15 targets respectively. The intersection of the targets obtained by these four calculation methods is the core targets.

### 2.8 Molecular docking

We used the original ligand of the target protein as a positive control for subsequent molecular docking. We downloaded the SDF format file of curcumin from PubChem and the SDF format file of the original ligand from the PDB database (https:// www.rcsb.org/). We convert the SDF format to mol2 format *via* OpenBabel-3.1.1. We import the mol2 format of the ligand into AutoDockTools 1.5.7 to set the torsion and output it as a pdbqt format file. The PDB file of the target protein is downloaded and the details of the protein are collected in the PDB database (Table 2). Proteins were dehydrated and de-liganded in PyMOL. Proteins were hydrogenated in AutoDockTools 1.5.7 and output as a file in pdbqt format. AutoDockTools 1.5.7 was restarted. The pdbqt files of the receptor and ligand are imported into AutoDockTools 1.5.7. When the docking box is constructed, the receptor protein is centered, the docking box completely covers the receptor protein and the ligand is located outside the docking box. The parameters of the docking box are collected (Table 3). Molecular docking is performed in AutoDockTools 1.5.7, and the magnitude of the binding energy reflects the possibility of binding between the receptor and ligand. The lower the binding energy, the higher the affinity between the receptor and the ligand. The lower the binding energy, the more stable the conformation of the receptor and the ligand. The binding energy of molecular docking is collected in AutoDockTools 1.5.7. The results of molecular docking are visualized in PyMOL.

**TABLE 2:** Details of the protein targets in the PDB database.

Targets	PDB ID	Method	Resolution (Å)	R-Value free	R-Value work	R-Value observed
CDK2	6Q4G	X-RAY DIFFRACTION	0.98	0.211	0.19	0.191
HSP90AA1	5J2X	X-RAY DIFFRACTION	1.22	0.206	0.199	0.199
AURKB	4AF3	X-RAY DIFFRACTION	2.75	0.264	0.205	0.208
CCNA2	6ATH	X-RAY DIFFRACTION	1.82	0.187	0.171	0.172
TYMS	3ED7	X-RAY DIFFRACTION	1.56	0.233	0.209	0.21
CHEK1	3PA3	X-RAY DIFFRACTION	1.40	0.213	0.193	—
AURKA	5DT0	X-RAY DIFFRACTION	2.15	0.225	0.21	0.211
DNMT1	5WVO	X-RAY DIFFRACTION	2.00	0.239	0.196	0.198
TOP2A	6ZY5	ELECTRON MICROSCOPY	3.60	—	—	—
TK1	2ORV	X-RAY DIFFRACTION	2.30	0.249	0.197	0.202

**TABLE 3:** Grid docking parameters in molecular docking.

Target name	PDB ID	Spacing (angstrom)	Center grid box		
			X center	Y Center	Z center
CDK2	6Q4G	0.547	1.857	−4.716	−14.407
DNMT1	5WVO	0.514	−12.638	−24.181	−2.143
TK1	2ORV	0.542	42.315	13.409	47.968
AURKB	4AF3	0.492	16.434	−17.71	−1.316
TYMS	3ED7	0.475	47.532	−8.991	38.219
CCNA2	6ATH	0.481	31.9	−6.634	45.838
CHEK1	3PA3	0.714	14.898	2.071	23.362
HSP90AA1	5J2X	0.397	1.4	14.792	29.787
AURKA	5DT0	0.536	−17.022	−32.344	7.974
TOP2A	6ZY5	1.000	162.757	162.756	143.014

### 2.9 External validation of core targets

#### 2.9.1 Gene expression levels of core targets

In the “Expression DIY” of GEPIA (http://gepia.cancer-pku.cn/), the mRNA expression levels and pathological stages of the core targets were verified (Tang et al., 2017). |log2FC|Cutoff:1, *p*-value Cutoff:0.01.

#### 2.9.2 Protein expression levels of core targets

To investigate the expression of core targets in CC tissues, we analyzed the core targets in the Human Protein Atlas database (https://www.proteinatlas.org/) (Uhlén et al., 2015). The protein expression levels of the core targets in CC tissues and normal colon tissues were compared.

#### 2.9.3 Genetic alterations in core targets

The colon Cancer (CPTAC-2 Prospective, Cell 2019) dataset containing 110 samples was selected for analysis in cBioPortal (https://www.cbioportal.org/) (Cerami et al., 2012). Information on the genetic alterations of the core targets was obtained.

#### 2.9.4 Immune cell infiltration of core targets

To elucidate the potential mechanisms of the immune microenvironment in CC, we entered the core targets into the TIMER database (https://cistrome.shinyapps.io/timer/) (Li et al., 2017) to explore the association between core targets and the level of immune infiltration.

## 3 Results

### 3.1 Targets of curcumin and CC

**FIGURE 2:**
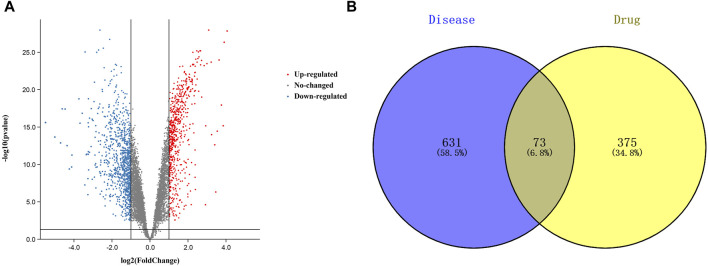
Targets relevant to the treatment of colon cancer. (**(A)**. Volcano plot of DEGs associated with colon cancer. **(B)**. Venn diagram showing the common part of curcumin and colon cancer).

A total of 448 curcumin action targets were obtained. A total of 1732 differentially expressed genes were screened from the GSE74602 dataset (Figure 2A). A total of 5102 targets were obtained by OMIM, DisGeNET and GeneCards. 1732 differentially expressed genes and 5102 targets were intersected to finally obtain 704 CC related targets.

### 3.2 Common target acquisition and PPI network construction

**FIGURE 3:**
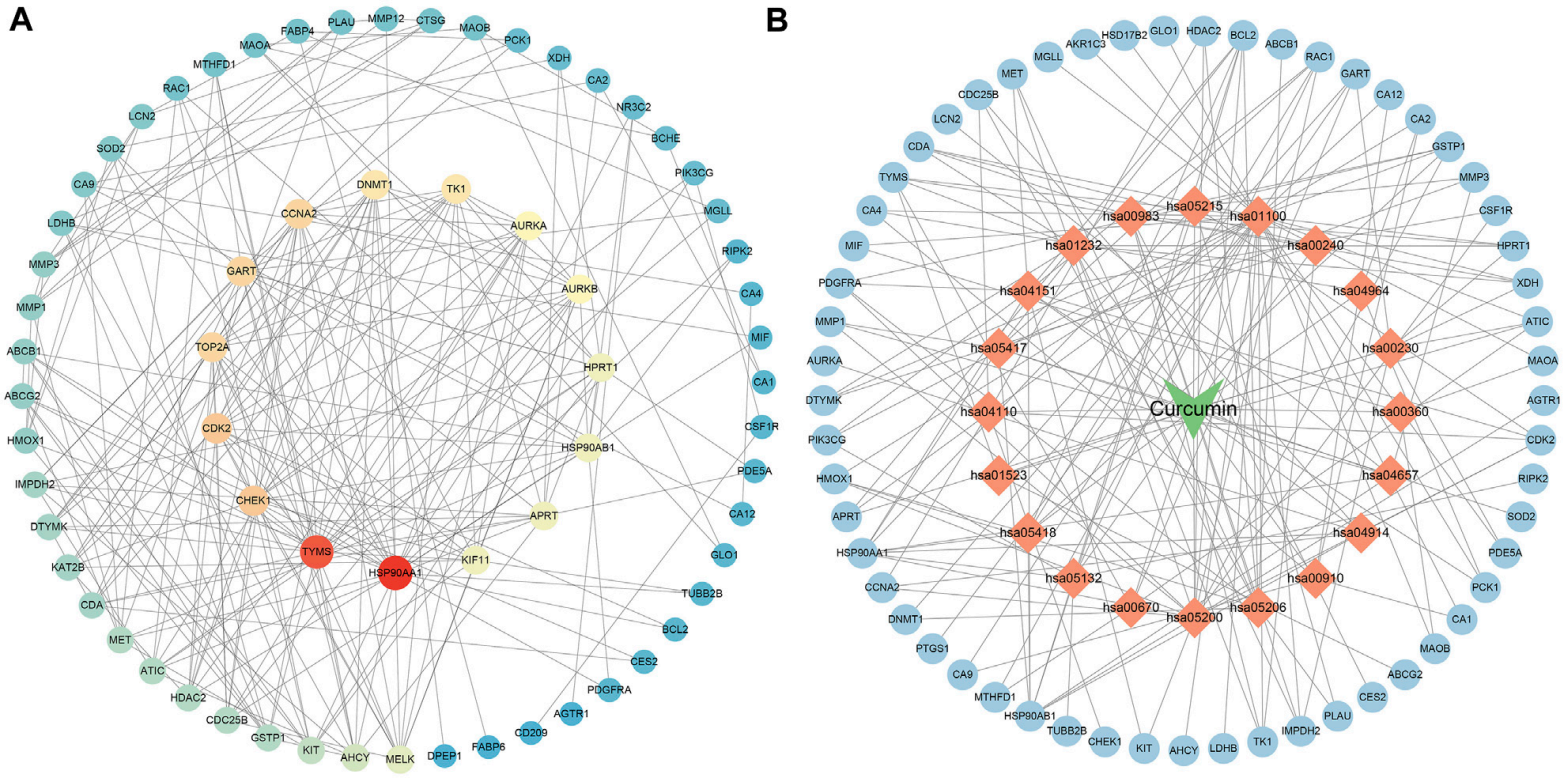
PPI network diagram (**(A)**. PPI network of potential targets for curcumin therapy of colon cancer. **(B)**. Drug-target-pathway network diagram. The blue circles represent targets, the orange diamonds are pathways, and the green triangles are curcumin).

The results of Venn diagram showed that 73 common targets were screened by matching 448 drug targets and 704 disease targets (Figure 2B). These 73 common targets were potential targets for curcumin in the treatment of CC. The 73 targets were imported into the STRING database to obtain the PPI network. The PPI network data were organized and visualized by Cytoscape 3.9.0, and 70 nodes and 230 edges were found in the PPI network. When the nodes are larger and darker, the degree of the node is larger (Figure 3A).

### 3.3 Results of GO and KEGG enrichment analysis

**FIGURE 4:**
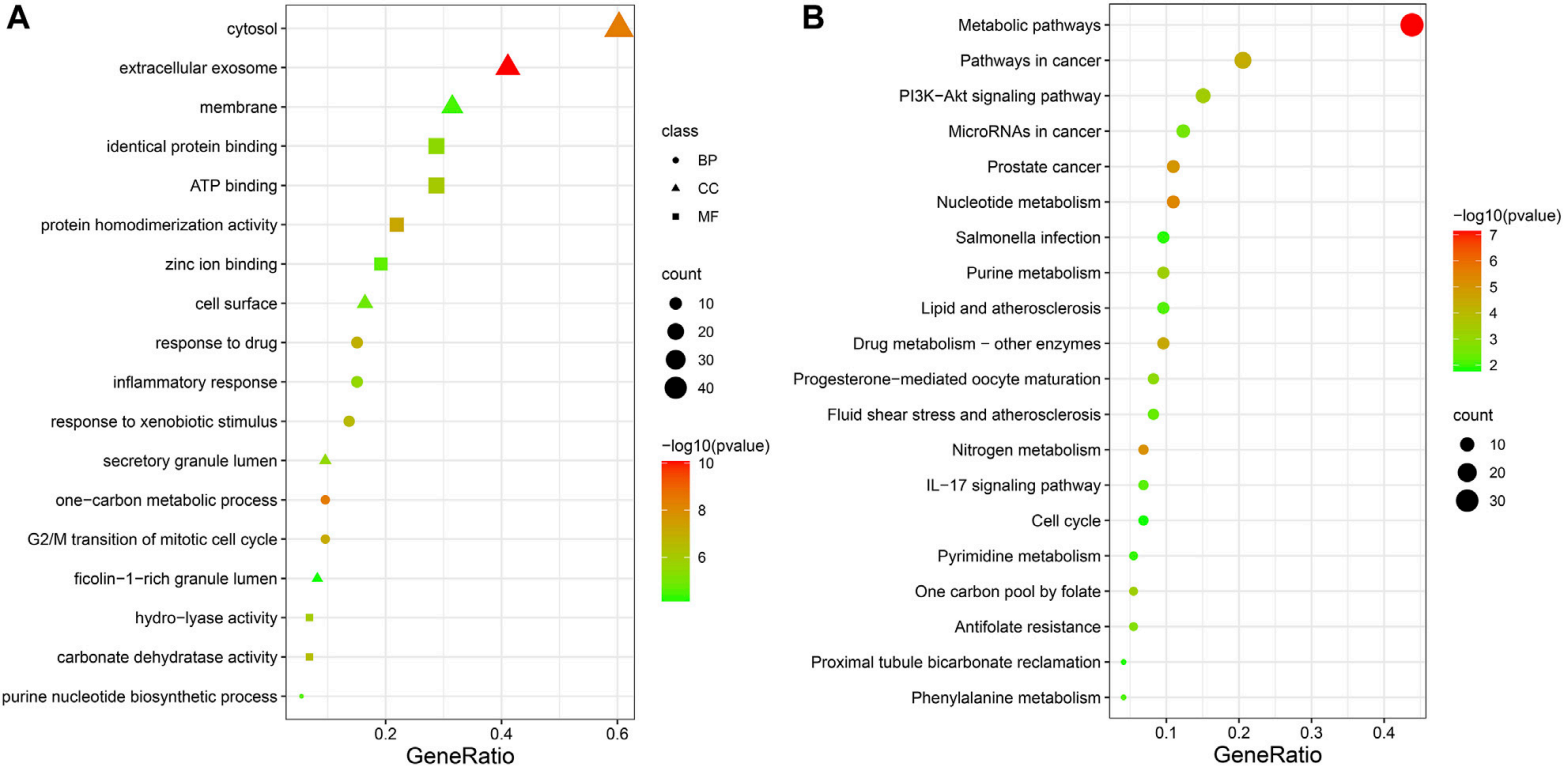
Bubble plot of enrichment analysis (**(A)**. GO functional enrichment analysis of curcumin in colon cancer. **(B)**. KEGG pathway enrichment analysis of curcumin in colon cancer).

**FIGURE 5:**
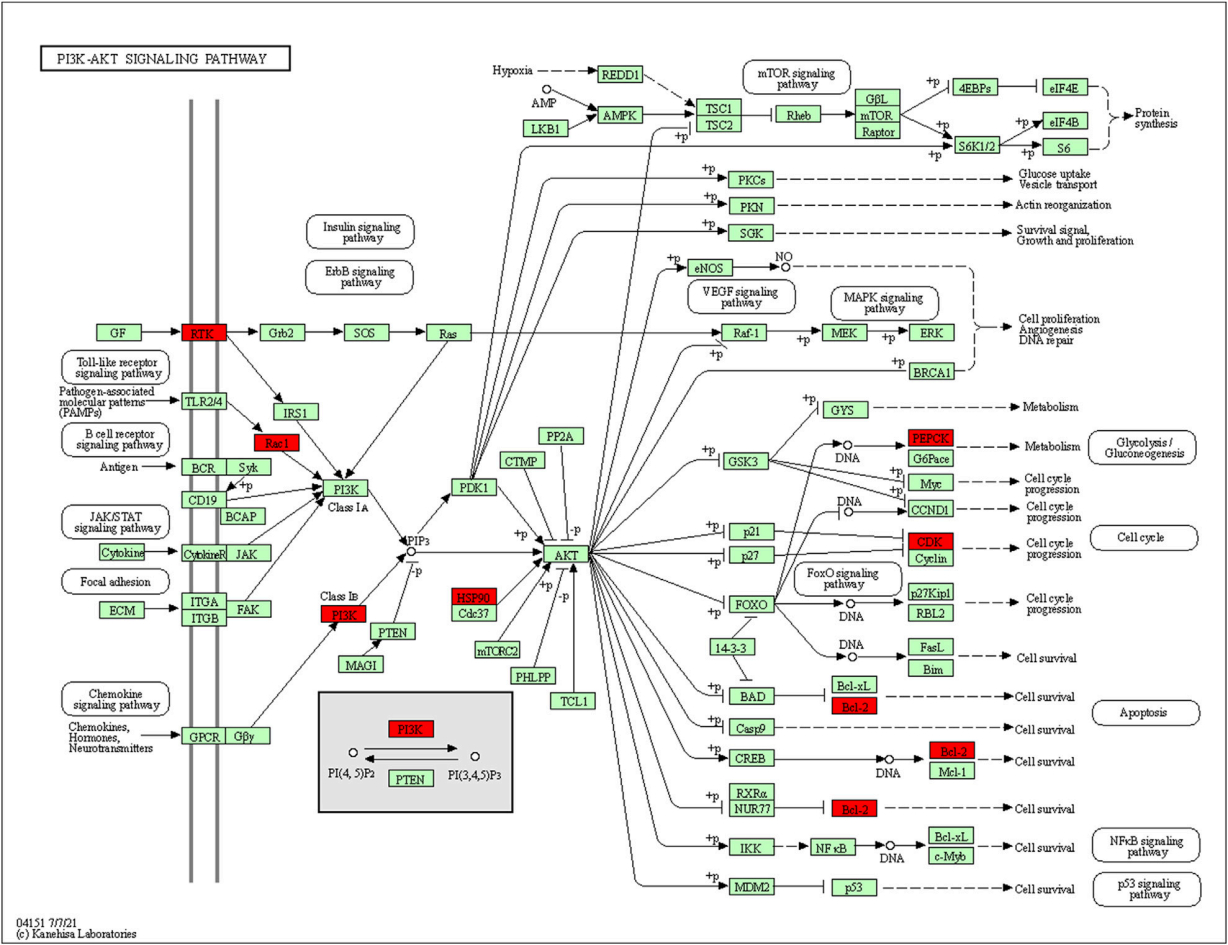
PI3K-Akt signaling pathway (red marks represent potential targets for curcumin intervention).

73 potential targets of curcumin for CC treatment were imported into DAVID for GO and KEGG enrichment analysis. The GO enrichment analysis yielded 256 entries, including BP (Biological Progress): 166, CC(celluar component):36 and MF (Molecular Function):54. The top 6 entries of each type of analysis were selected for visualization in Bioinformatics (Figure 4A). 73 targets were involved in biological processes mainly one-carbon metabolic process,G2/M transition of mitotic cell cycle, response to drug, response to xenobiotic stimulus, inflammatory response, purine nucleotide biosynthetic process, etc. It mainly functions in the extracellular exosome, cytosol, secretory granule lumen, cell surface, membrane, ficolin-1-rich granule lumen, etc. The main molecular functions involved are protein homodimerization activity, carbonate dehydratase activity, ATP binding, hydro-lyase activity, identical protein binding, zinc ion binding, etc. KEGG pathway enrichment analysis screened 34 signaling pathways and visualized the top 20 pathways (Figure 4B), which mainly involved Metabolic pathways, Nucleotide metabolism, Nitrogen metabolism, Drug metabolism - other enzymes, Pathways in cancer, PI3K-Akt signaling pathway, etc. The PI3K-Akt signaling pathway is more important and this pathway was selected for mapping (Figure 5). The red markers in the figure represent potential targets for curcumin intervention.

### 3.4 Drug-target-pathway network construction

The top 20 KEGG pathways were imported into Cytoscape to construct a drug-target-pathway network (Figure 3B). The blue circles are targets, the orange diamonds are pathways, and the green triangles are curcumin. The results showed that curcumin exerts its effects in treating CC through multiple targets and multiple signaling pathways.

### 3.5 Molecular docking validation of curcumin and core targets

**FIGURE 6:**
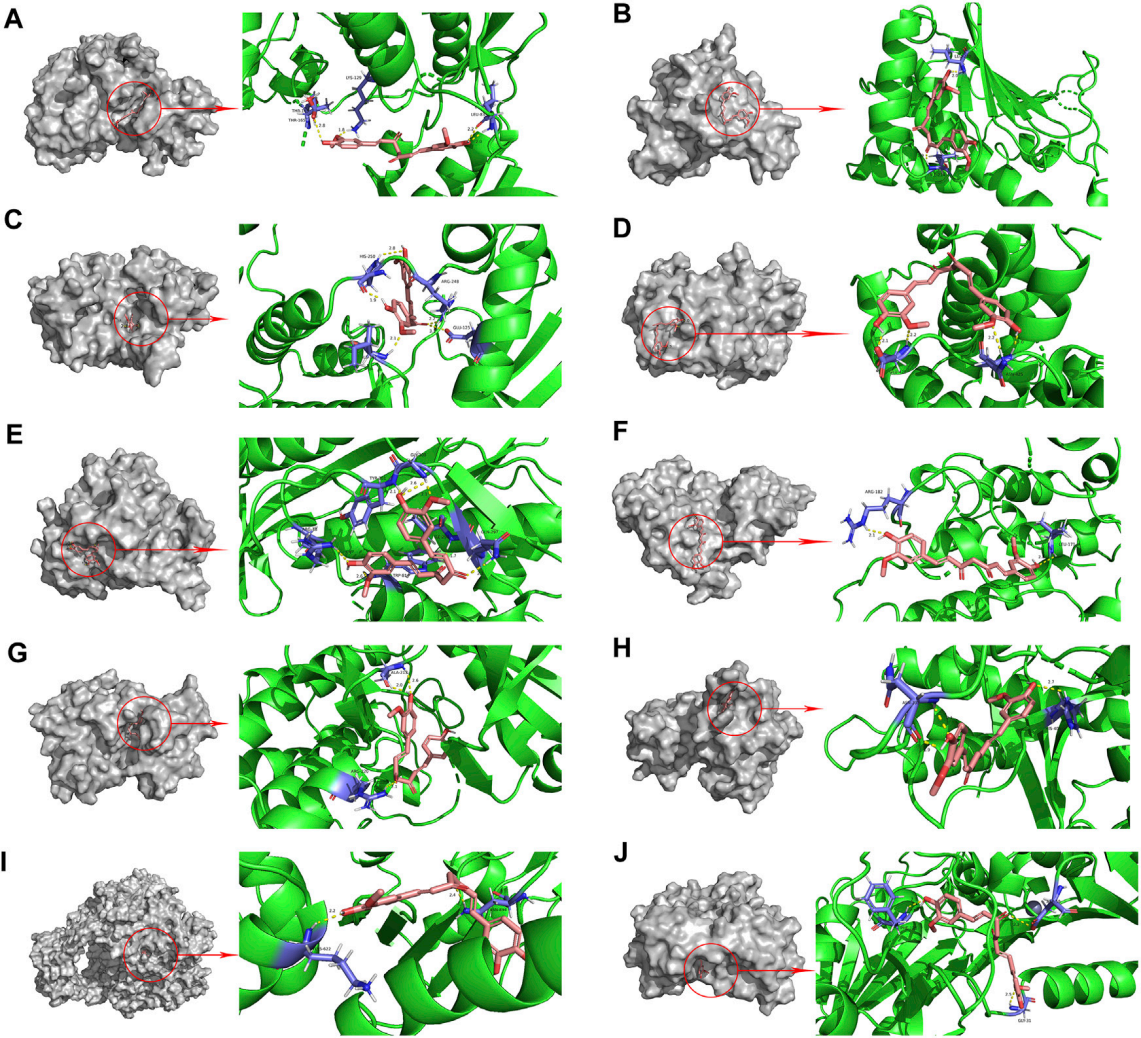
Molecular docking pattern of curcumin and core target protein. (**(A)**. Curcumin-CDK2, **(B)**. Curcumin-HSP90AA1, **(C)**. Curcumin-AURKB, **(D)**. Curcumin-CCNA2, **(E)**. Curcumin-TYMS, **(F)**. Curcumin-CHEK1, **(G)**. Curcumin-AURKA, **(H)**. Curcumin-DNMT1, **(I)**. Curcumin-TOP2A, **(J)**. Curcumin-TK1).

**FIGURE 7:**
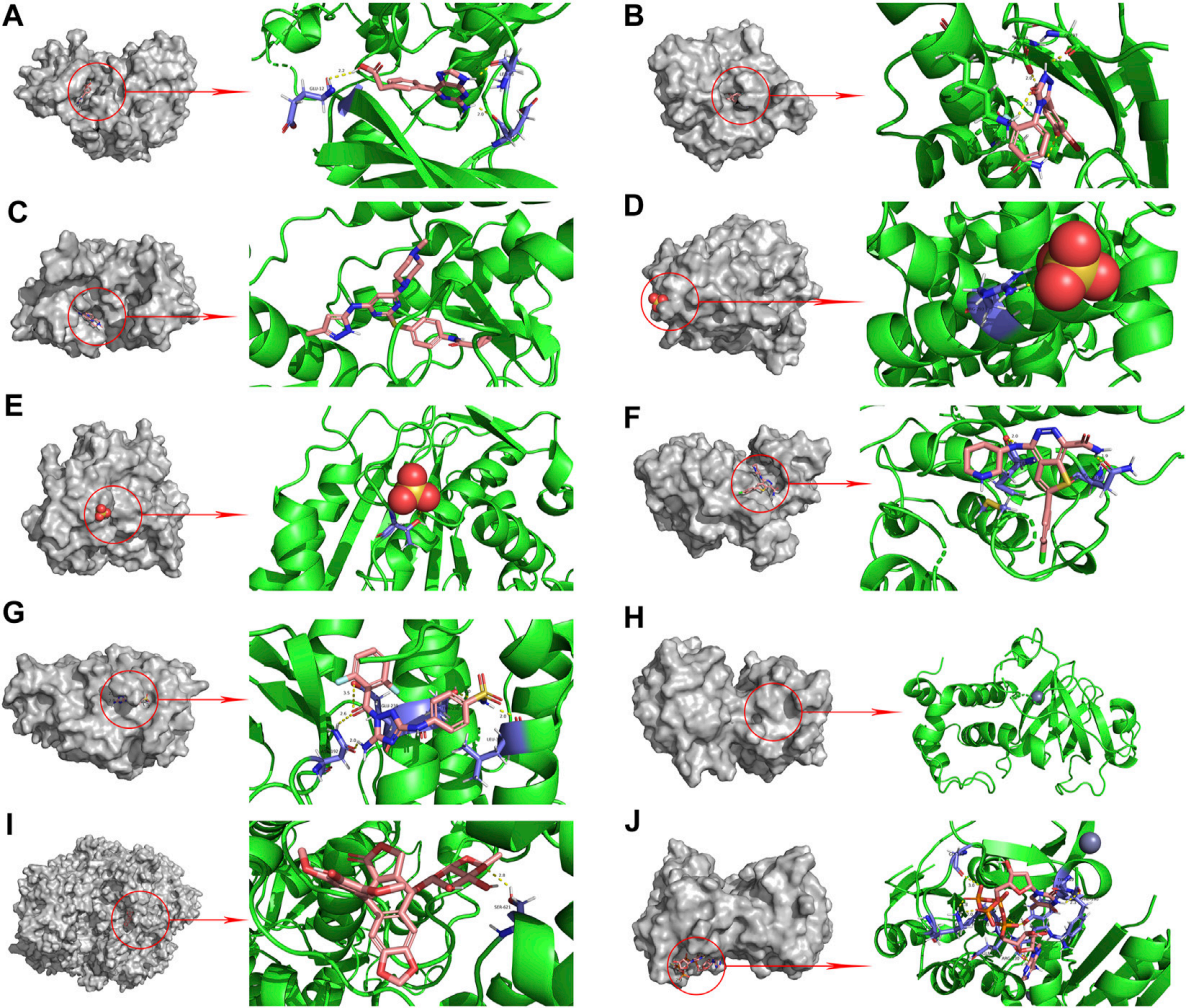
Molecular docking pattern of original ligand and core target protein. (**(A)**. HJK-CDK2, **(B)**. 6DL-HSP90AA1, **(C)**. VX6-AURKB, **(D)**. SO4-CCNA2, **(E)**. SO4-TYMS, **(F)**. C70-CHEK1, **(G)**. SKE-AURKA, **(H)**. ZN-DNMT1, **(I)**. EVP-TOP2A, **(J)**. 4TA-TK1).

The PPI network diagram of potential targets (Figure 3A) was analyzed by CytoHubba, and 10 core targets were selected (Table 4). The molecular docking results showed that the binding energies between curcumin and the target proteins were all less than 0. Curcumin is tightly linked to amino acid residues through hydrogen bonds. The binding energy, amino acid residues and hydrogen bonds are collected (Table 5). The binding energies of curcumin to CDK2, DNMT1 and TK1 were all smaller than those of the positive control, suggesting that curcumin has a stronger binding capacity to these target proteins than the positive control. The binding energies of curcumin with CCNA2, TYMS, TOP2A, HSP90AA1, AURKB, CHEK1, and AURKA were all close to those of the positive control, which indicates that the binding ability between curcumin and these target proteins was close to that of the positive control. In summary, we can know that the results of molecular docking are plausible and true, that curcumin binds strongly to core target proteins, and that curcumin may exert anticancer effects by binding to core target proteins. The results of molecular docking are visualized (Figures 6, 7).

**TABLE 4:** 10 Hub genes identified using 4 different algorithms in the Cytohubba plugin.

Rank	Gene symbol	Full name	Score
1	*CDK2*	Cyclin-dependent kinase 2	141986
2	*TOP2A*	DNA topoisomerase 2-alpha	141984
3	*CCNA2*	Cyclin-A2	141984
4	*AURKA*	Aurora kinase A	141120
5	*AURKB*	Aurora kinase B	136104
6	*CHEK1*	Serine/threonine-protein kinase Chk1	101668
7	*TYMS*	Thymidylate synthase	86224
8	*TK1*	Thymidine kinase, cytosolic	40584
9	*DNMT1*	DNA (cytosine-5)-methyltransferase 1	15851
10	*HSP90AA1*	Heat shock protein HSP 90-alpha	15183

**TABLE 5:** Basic information on the molecular docking of curcumin and target proteins.

Molecular name	Targets	PDB ID	Residue involved in H bonding	H-bond length (Å)	Binding energy (kcal/Mol)
Curcumin	CDK2	6Q4G	THR-165; LYS-129; LEU-83	2.8; 1.8; 2.2,2.0	−6.63
Curcumin	DNMT1	5WVO	ASN-392; HIS-405	2.1,1.9; 2.7	−6.62
Curcumin	TK1	2ORV	PHE-128; ASP-58; GLY-31	2.3; 3.3; 2.5	−6.18
Curcumin	AURKB	4AF3	HIS-250; ARG-248; GLU-125; ILE-197	2.8,1.9; 2.2; 3.1; 2.1	−5.78
Curcumin	TYMS	3ED7	GLY-300; TYR-301; ARG-78; ILE-298; GLN-297; TRP-81	2.6,2.1; 2.1; 2.3; 1.7; 2.1; 2.6	−5.56
Curcumin	CCNA2	6ATH	GLU-428; ASN-425	2.1,2.2; 2.2,2.0	−5.32
Curcumin	CHEK1	3PA3	ARG-182; LEU-171	2.1; 2.8	−4.79
Curcumin	HSP90AA1	5J2X	LEU-220; ILE-214	2.0; 1.9	−4.58
Curcumin	AURKA	5DT0	ALA-213; ARG-220	2.0,2.6; 2.1	−4.51
Curcumin	TOP2A	6ZY5	LYS-622; ASN-894	2.2; 2.4	−3.07
HJK	CDK2	6Q4G	GLU-12; LEU-83; GLU-81	2.2; 2.0; 2.0	−6.46
ZN	DNMT1	5WVO	—	—	−1.00
4 TA	TK1	2ORV	LYS-170; GLY-167; GLN-129; ARG-130; PHE-128; TYR-189; PHE-190	1.8; 2.0; 3.0; 2.6; 2.4; 3.5; 2.5; 2.3	−4.82
VX6	AURKB	4AF3	—	—	−8.12
SO4	TYMS	3ED7	THR-306	3.2	−5.76
SO4	CCNA2	6ATH	ARG-293	2.0	−5.89
C70	CHEK1	3PA3	MET-167; ASN-165	2.0; 1.9	−7.01
6DL	HSP90AA1	5J2X	ASP-93; GLY-97; LYS-58; ASN-51	2.0; 2.1; 2.2; 2.4	−7.62
SKE	AURKA	5DT0	GLU-239; ASN-192; THR-238; LEU-378	3.5,2.6; 2.0; 2.0; 2.0	−6.16
EVP	TOP2A	6ZY5	SER-621	2.8	−3.32

### 3.6 External validation of core targets

#### 3.6.1 The mRNA expression levels of core targets

**FIGURE 8:**
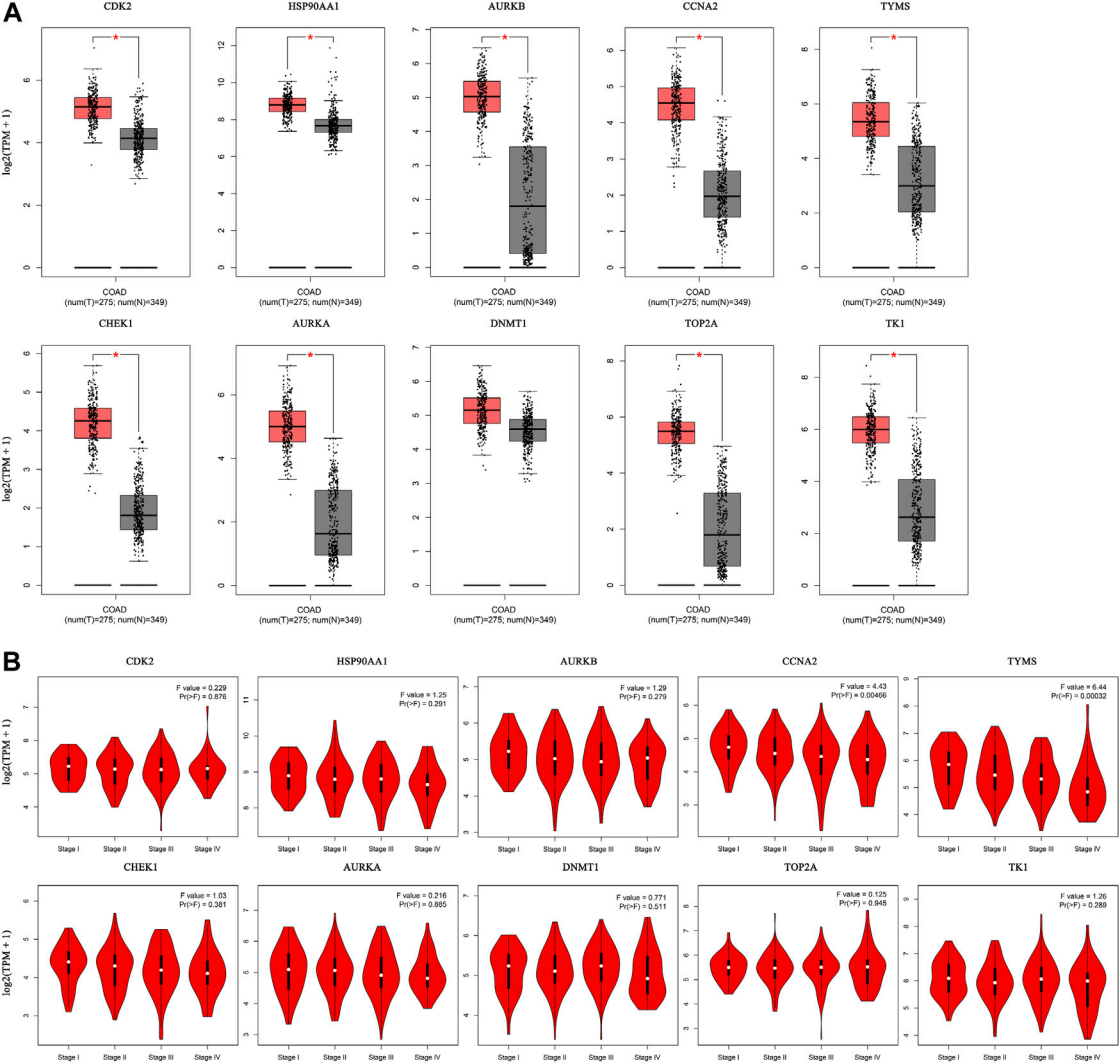
Hub gene expression in the GEPIA database. (**(A)**. Box plot of hub gene mRNA expression levels in the GEPIA database. Red represents tumor tissues and gray represents normal tissues. **(B)**. Stage diagram of hub gene mRNA expression levels and pathological stages in the GEPIA database).

The expression of the core targets was different in CC tissues and normal tissues. The mRNA levels of CDK2, HSP90AA1, AURKB, CCNA2, TYMS, CHEK1, AURKA, TOP2A, and TK1 were significantly higher in CC than in normal tissues (*p* <0.01) (Figure 8A). In addition, we analyzed the relationship between the mRNA levels of core targets and the pathological stage of CC. The results showed that the mRNA levels of CCNA2 and TYMS significantly changed with pathological stage (*p* <0.01) (Figure 8B).

#### 3.6.2 Protein expression levels of core targets

**FIGURE 9:**
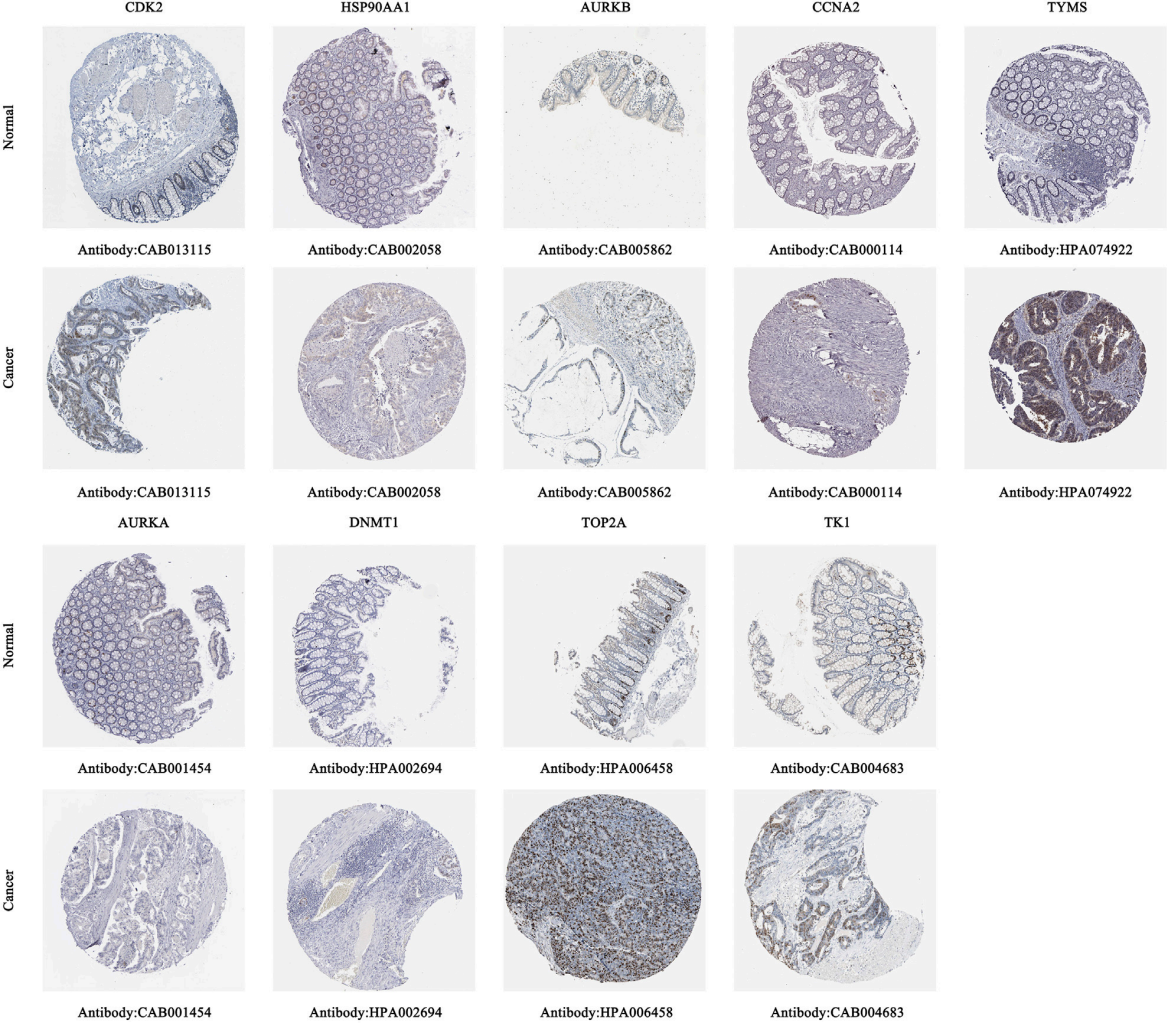
Immunohistochemical images of hub gene protein expression levels in the HPA database.

Immunohistochemical staining images in the HPA database were analyzed to observe the expression levels of core target proteins in CC. We found elevated expression levels of CDK2, HSP90AA1, AURKB, CCNA2, TYMS, AURKA, DNMT1, TOP2A, and TK1 in CC tissues compared with normal colon tissues (Figure 9). No immunohistochemical data for CHEK1 were found in the HPA database.

#### 3.6.3 Genetic alterations in core targets

**FIGURE 10:**
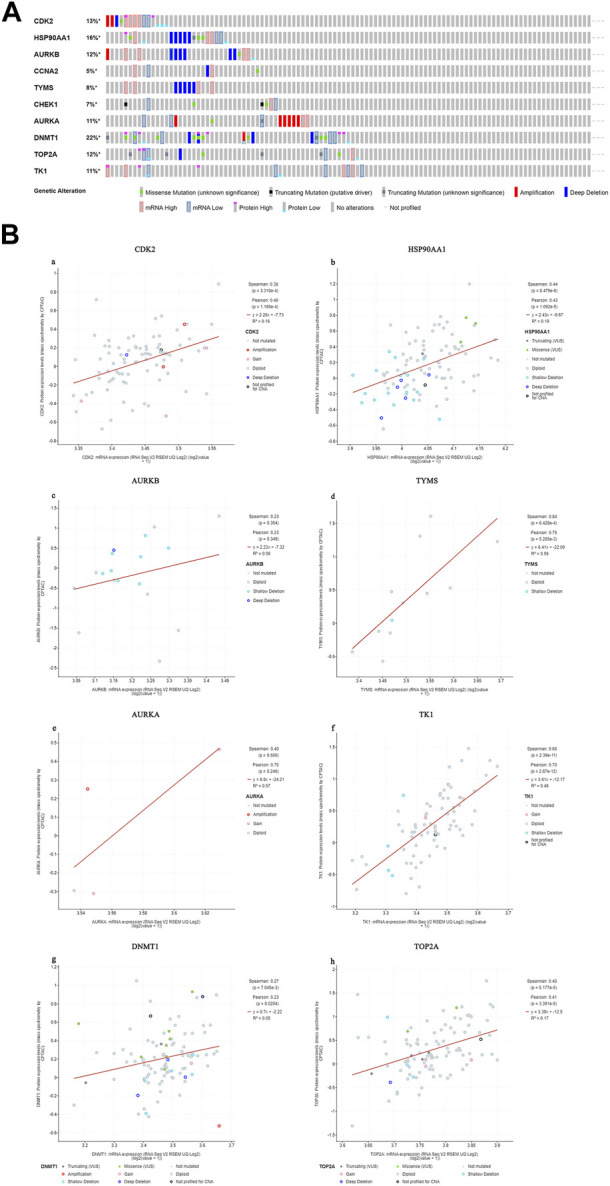
Genetic information of hub targets. (**(A)**. Data showed that 57 of 110 patients (52%) had genetic mutations in these targets. **(B)**. The diagram shows the correlation between the mRNA and protein levels of **(a)** CDK2, **(b)** HSP90AA1, **(c)** AURKB, **(d)** TYMS, **(e)** AURKA, **(f)** TK1, **(g)** DNMT1, **(h)** TOP2A).

We found that 57 of 110 CC patients (52%) had genetic mutations in these targets (Figure 10A). We found a positive correlation between protein expression and mRNA levels of the core targets (Figure 10B), and no correlation data were found for CCNA2 and CHEK1.

#### 3.6.4 Immune cell infiltration of core targets

**FIGURE 11:**
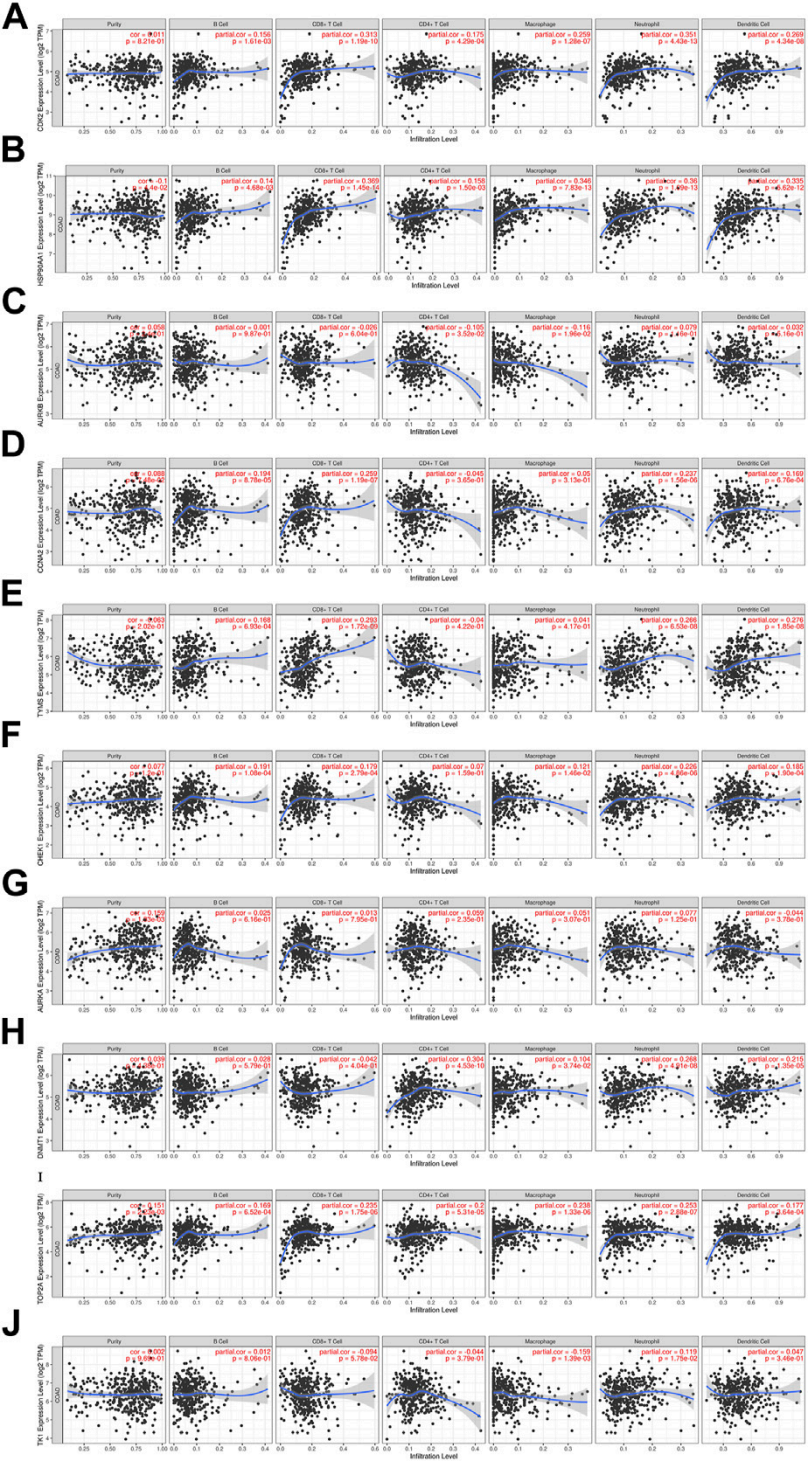
Relationship between differentially expressed core targets and immune cell infiltration. (**(A)**. CDK2, **(B)**. HSP90AA1, **(C)**. AURKB, **(D)**. CCNA2, **(E)**. TYMS, **(F)**. CHEK1, **(G)**. AURKA, **(H)**. DNMT1, **(I)**. TOP2A, **(J)**. TK1).

The relationship between core targets and immune cell infiltration was analyzed. The results showed that the expression of HSP90AA1 was positively correlated with the infiltration of B cells, CD8^+^ T cells, CD4^+^ T cells, macrophages, neutrophils and dendritic cells. The expression of HSP90AA1 was negatively correlated with purity. The expression of AURKB and TK1 were positively correlated with infiltration of purity, B cells, neutrophils and dendritic cells. The expression of AURKB and TK1 were negatively correlated with infiltration of CD8^+^ T cells, CD4^+^ T cells and macrophages. The expression of CCNA2 was positively correlated with infiltration of purity, B cells, CD8^+^ T cells, macrophages, neutrophils, and dendritic cells. The expression of CCNA2 was negatively correlated with infiltration of CD4^+^ T cells. The expression of TYMS was positively correlated with infiltration of B cells, CD8^+^ T cells, macrophages, neutrophils and dendritic cells. The expression of TYMS was negatively correlated with infiltration of purity and CD4^+^ T cells. The expression of AURKA was positively correlated with infiltration of purity, B cells, CD8^+^ T cells, CD4^+^ T cells, macrophages and neutrophils. The expression of AURKA was negatively correlated with infiltration of dendritic cells. The expression of DNMT1 was positively correlated with infiltration of purity, B cells, CD4^+^ T cells, macrophages, neutrophils and dendritic cells. The expression of DNMT1 was negatively correlated with infiltration of CD8^+^ T cells. The expression of CDK2, CHEK1 and TOP2A were positively correlated with the infiltration of purity, B cells, CD8^+^ T cells, CD4^+^ T cells, macrophages, neutrophils and dendritic cells (Figure 11). We analyzed the clinical significance of core targets and immune cell infiltration in CC using a Cox proportional risk model. The results showed that age, CD8^+^ T cells, CDK2 and CCNA2 were significantly associated with clinical outcomes in patients with CC (Table 6).

**TABLE 6:** Cox proportional hazard model for hub genes and tumor-infiltrating immune cells.

	Coef	HR	95%CI_l	95%CI_u	p.Value	Sig
Age	0.022	1.022	1.000	1.044	0.048	*
gendermale	0.433	1.542	0.904	2.630	0.112	
raceBlack	−0.321	0.725	0.087	6.035	0.766	
raceWhite	−0.251	0.778	0.098	6.178	0.813	
Purity	0.158	1.172	0.263	5.214	0.835	
B_cell	2.553	12.841	0.017	9610.271	0.450	
CD8_Tcell	−5.940	0.003	0.000	0.630	0.034	*
CD4_Tcell	−2.035	0.131	0.000	68.843	0.525	
Macrophage	4.588	98.334	0.317	30493.335	0.117	
Neutrophil	0.868	2.381	0.000	117815.288	0.875	
Dendritic	0.099	1.104	0.015	79.585	0.964	
CDK2	1.086	2.963	1.183	7.421	0.020	*
HSP90AA1	−0.020	0.980	0.565	1.698	0.942	
AURKB	0.204	1.227	0.682	2.207	0.496	
CCNA2	−0.705	0.494	0.253	0.964	0.039	*
TYMS	−0.246	0.782	0.486	1.257	0.310	
CHEK1	−0.113	0.893	0.426	1.875	0.765	
AURKA	−0.173	0.841	0.479	1.478	0.548	
DNMT1	−0.246	0.782	0.409	1.496	0.458	
TOP2A	−0.034	0.966	0.544	1.716	0.907	
TK1	0.497	1.644	0.924	2.926	0.091	

Notes: *p < 0.05.

## 4 Discussion

CC is a common malignant tumor of the gastrointestinal tract, and most patients have already metastasized at the time of diagnosis (Liu et al., 2019; Taieb and Gallois, 2020), and it is easy to recur even through surgical treatment. Although chemotherapeutic drugs have obvious anti-tumor effects, they can also cause serious adverse effects while killing tumor cells. Curcumin, the active ingredient of turmeric, has been shown to have strong antitumor effects in clinical trials against cancers such as liver, colon, and breast cancers (Lee et al., 2009). However, the mechanism of action of traditional Chinese medicine for disease treatment is multiple targets and multiple pathways, so we need to apply big data to explore the targets and pathways of curcumin and CC. The aim of this study was to explore the potential molecular mechanism of the inhibitory effect of curcumin on CC using network pharmacology combined with bioinformatics, and to provide some theoretical basis for the clinical application of curcumin and the study of CC.

According to the GO enrichment results, curcumin mainly acts in the extracellular exosome, cytosol, secretory granule lumen, cell surface, membrane,ficolin-1-rich granule lumen and other sites. The molecular functions involved are protein homodimerization activity, carbonate dehydratase activity, ATP binding,hydro-lyase activity, identical protein binding, zinc ion binding, etc. In addition, we found that curcumin exerts its effect on the treatment of CC by affecting biological processes such as one-carbon metabolic process,G2/M transition of mitotic cell cycle, response to drug, response to xenobiotic stimulus, inflammatory response, purine nucleotide biosynthetic process, etc.

The KEGG enrichment results showed that many disease pathways that were not relevant to this study were enriched, probably because the same molecular targets exist in the development of different diseases. So we selected signaling pathways that are closely related to CC for analysis. We found that the therapeutic effect of curcumin on CC may be produced by regulating PI3K-Akt signaling pathway, IL-17 signaling pathway and Cell cycle. PI3K-Akt signaling pathway is closely related to the progression of many cancers. During tumor progression, the PI3K-Akt signaling pathway can be activated by multiple types of cellular stimulation or toxic injury, regulating essential cellular functions such as transcription, translation, proliferation, growth and survival. Binding of growth factors to their receptor tyrosine kinase (RTK) stimulates the class la Pl3K subtype, and binding of chemokines, hormones and neurotransmitters to G protein-coupled receptors (GPCR) stimulates the class lb Pl3K subtype. PI3K catalyzes the production of phosphatidylinositol-3,4,5-trisphosphate (PIP3) in cell membranes. PIP3 acts as a second messenger and helps to activate Akt. Akt can regulate a number of key cellular processes by phosphorylating substrates for apoptosis, protein synthesis, metabolism and the cell cycle, promoting cancer cell growth and survival (Chang et al., 2003; Lee et al., 2008).

The interleukin 17 (IL-17) family is a subgroup of cytokines consisting of IL-17A-F that plays a key role in both acute and chronic inflammatory responses. Studies have shown that when IL-17 signaling pathway expression is inhibited, the number of colorectal tumors is reduced and cancer cells have a reduced ability to proliferate (Pan et al., 2022). Cell cycle regulation is inextricably linked to apoptosis. Cell cycle arrest can occur when the cell cycle is depleted or when DNA damage is severe. When cell cycle arrest is irreversible, cells initiate the apoptotic program and apoptosis occurs. Studies have shown that cell cycle arrest can be induced in human CC cells by elevating the expression of cell cycle inhibitory proteins and decreasing the expression of cell cycle progressive proteins, producing an anti-cancer effect (Choi et al., 2019).

The targets of curcumin and CC were taken to intersect and 73 potential targets of curcumin for the treatment of CC were obtained. The top 10 core targets (CDK2, HSP90AA1, AURKB, CCNA2, TYMS, CHEK1, AURKA, DNMT1, TOP2A, and TK1) were further screened. CDK2 is a central factor in the oncogenic signaling pathway and has an important role in the tumor process. When CDK2 is inhibited, cancer cells undergo apoptosis and growth arrest (Barrière et al., 2007). HSP90AA1 is a molecular chaperone that promotes the maturation, structural maintenance and proper regulation of specific target proteins involved in cell cycle control and signal transduction. In colorectal cancer, there is a positive correlation between high expression of HSP90AA1 and poorer prognosis of patients (Zhang et al., 2019). Studies have shown that HSP90AA1 enhances the proliferation and invasion of tumor cells, further worsening the disease, and that HSP90AA1 may be a potential target for the treatment of cancer (Wu et al., 2017; Tian et al., 2019).

AURKB is involved in the bipolar attachment of spindle microtubules to kinetochores and is a key regulator of cytokinesis onset during mitosis. Histone H3 on serine 10 and serine 28 can be phosphorylated by AURKB, which is associated with chromosome number stability and chromatin condensation during mitosis (Goldenson and Crispino, 2015). AURKB is highly expressed in tumors, and AURKB overexpression is associated with poor prognosis (Tanaka et al., 2008; Hegyi et al., 2012). CCNA2 is a cell cycle protein that controls the cell cycle by forming specific protein kinase complexes with protein kinases. Overexpression of CCNA2 is associated with poorer OS and DFS in pancreatic ductal adenocarcinoma, and CCNA2 overexpression is associated with disease progression in pancreatic ductal adenocarcinoma (Dong et al., 2019).

TYMS is highly expressed in patients with colorectal cancer and non-small cell lung cancer, and patients with lower TYMS mRNA levels have higher survival rates than those with higher expression (Sun et al., 2015; Jiang et al., 2019). CHEK1 has some anti-apoptotic ability, and a positive correlation between CHEK1 overexpression and tumor malignancy and poorer prognosis has been noted in colorectal cancer (Gali-Muhtasib et al., 2008). AURKA, an oncogene, is highly expressed in cancer patients (Umene et al., 2013; Kivinummi et al., 2017). AURKA exerts its cancer-inducing effects through the Wnt and MAPK signaling pathways (Jacobsen et al., 2018). During DNA replication, DNMT1 is a DNA methyltransferase responsible for maintaining the methylation state of DNA. DNMT1 is highly expressed in cancer (Hino et al., 2009; Wu et al., 2011), and inhibition of DNMT1 slows the progression of cancer (Sun et al., 2017; Wang and Li, 2017; Han et al., 2018).

TOP2A is a key enzyme that alters the topology of DNA by binding to double-stranded DNA molecules. The proliferation and invasion of CC cells can be inhibited and apoptosis can be induced by down-regulating the expression of TOP2A (Zhang et al., 2018). TK1 is a cell cycle regulatory enzyme that plays an important role in nucleotide metabolism. It has been found that TK1 expression is high in cancer patients and high serum TK1 levels are usually associated with cancer stage and increased tumor size (Wu et al., 2013). Serum TK1 expression has been used as a prognostic tool to monitor response to chemotherapy or surgery (Zhang et al., 2006).

The molecular docking results showed that curcumin spontaneously bound to core target proteins. It suggested that curcumin could regulate the biological activity of CC-related targets. The reliability of the core targets of curcumin for CC treatment screened by network pharmacology was verified.

## 5 Conclusion

In summary, this study systematically illustrated the potential mechanism of curcumin for the treatment of CC through network pharmacology and molecular docking. Curcumin plays an important role in the treatment of CC through multiple targets and pathways after entering the body. The results showed that curcumin could exert anti-cancer effects by binding to CDK2, HSP90AA1, AURKB, CCNA2, TYMS, CHEK1, AURKA, DNMT1, TOP2A, and TK1. Curcumin interferes with tumor cell proliferation and apoptosis by regulating PI3K-Akt signaling pathway,IL-17 signaling pathway, Cell cycle and other signal transduction pathways. These reflect the anti-CC mechanism of curcumin. Due to the poor accuracy and completeness of the database during the network pharmacology study, biological experiments and extensive evidence-based medical validation are still needed at a later stage to ensure the reliability of the study results. Our study provides a new basis for further exploration of the role of curcumin in the treatment of CC and subsequent experimental validation.

## Data Availability

The original contributions presented in the study are included in the article/supplementary material, further inquiries can be directed to the corresponding authors.
